# Supplementary material for: miRNA changes with ageing and caloric restriction in male rat skeletal muscle: potential roles in muscle cell function
Source: Biogerontology. 2025 Nov 11;26(6):202. doi: 10.1007/s10522-025-10336-6 (PMC12605627; doi:10.1007/s10522-025-10336-6)
Supplement: Supplementary file 1 — Supplementary file1 (PDF 412 KB) [file 10522_2025_10336_MOESM1_ESM.pdf]

Additional File:

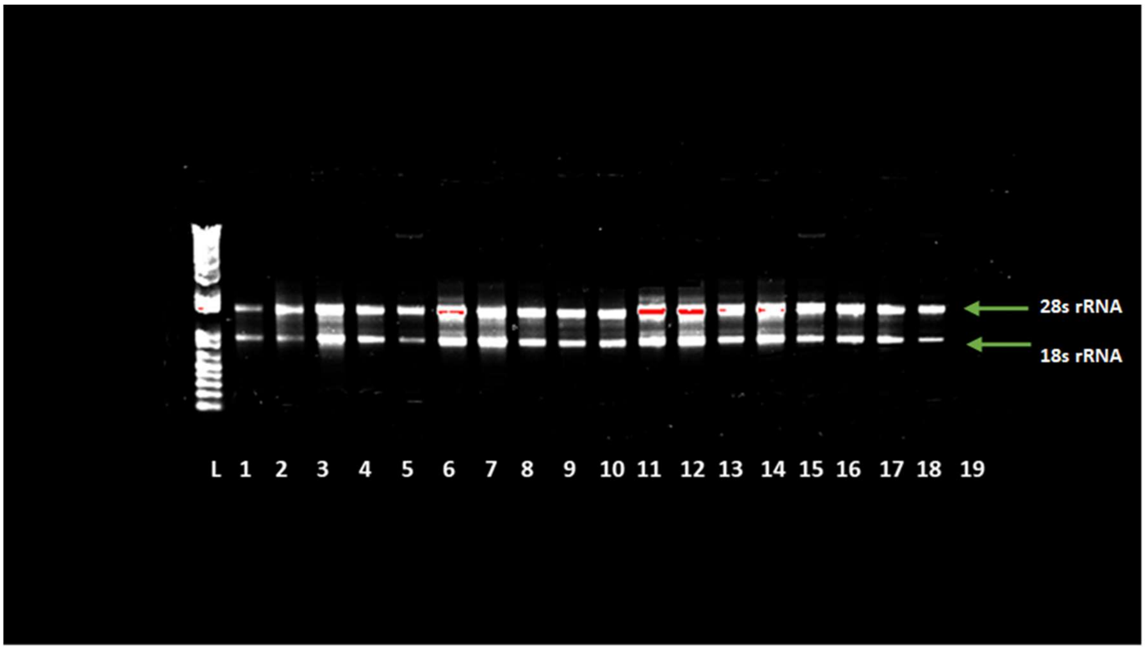

Figure S1.*Quality analysis of RNA samples as observed in 1% agarose gel. All RNAs from all samples were intact and good quality. Lane L represents DNA ladder, Lanes 1-18 represents RNA samples and Lane 19 represents negative control.*

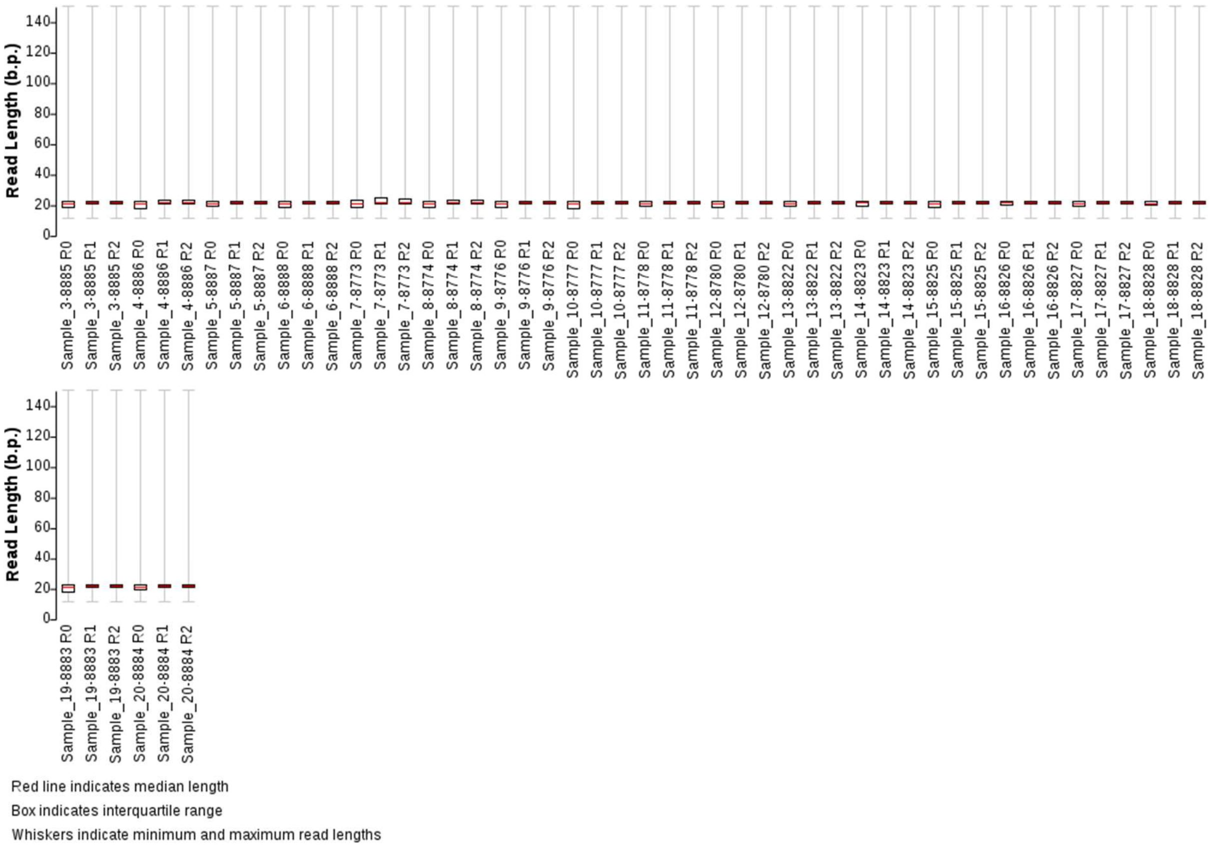

Figure S2. Read-length distribution of sequenced miRNAs in rat skeletal muscle.
Boxplots show the size distribution of processed miRNA reads after adapter trimming and removal of sequences shorter than 12 nucleotides. The majority of reads were centred at ~22 nucleotides, consistent with the expected size of mature miRNAs, confirming successful enrichment of small RNAs.

Mapping stats from miRDeep2 report:

Sample	Total reads	Mapped reads	Unmapped reads	% mapped	% unmapped
1	68300149	42713497	25586652	62.538	37.462
2	76140593	57291531	18849062	75.244	24.756
3	42556774	26754778	15801996	62.868	37.132
4	56843058	32273370	24569688	56.776	43.224
5	72227232	52068357	20158875	72.09	27.91
6	63120367	41188183	21932184	65.253	34.747
7	78142797	39970578	38172219	51.151	48.849
8	81631308	47633265	33998043	58.352	41.648
9	77175505	52947208	24228297	68.606	31.394
10	78604687	51606842	26997845	65.654	34.346
11	62934384	51039399	11894985	81.099	18.901
12	82202441	61850299	20352142	75.241	24.759
13	79788363	62919139	16869224	78.858	21.142
14	73895774	54202542	19693232	73.35	26.65
15	52013579	36957975	15055604	71.054	28.946
16	86913934	64157095	22756839	73.817	26.183
17	57756588	38995574	18761014	67.517	32.483
18	76276799	53717880	22558919	70.425	29.575

Table S1. Mapping statistics for miRNA sequencing.
Summary of sequencing quality metrics for 18 skeletal muscle samples. The table shows the total number of reads obtained per sample, the number of reads successfully mapped to known miRNAs, the number of unmapped reads, and the corresponding mapping percentages. These data confirm that the majority of reads mapped to known miRNAs across all samples, supporting the integrity of the extracted small RNAs.
